# Reinforced Granular Hydrogels Scaffolds with Tunable Physicochemical Properties for Advanced Skin Tissue Engineering

**DOI:** 10.1002/advs.202415634

**Published:** 2025-05-05

**Authors:** Jing Zhang, Yijia Wang, Yue Liu, Guanfu Wu, Guilong Lu, Yue Li, Yu Shen, Caifeng Wang, Mehdi Khanmohammadi, Wojciech Święszkowski, Jing Wang, Ziyi Yu

**Affiliations:** ^1^ State Key Laboratory of Materials‐Oriented Chemical Engineering College of Chemical Engineering Nanjing Tech University 30 Puzhu South Road Nanjing 211816 P. R. China; ^2^ Reproductive Medicine Center Zhongshan Hospital Fudan University Shanghai 200032 P. R. China; ^3^ Faculty of Materials Science and Engineering Warsaw University of Technology Warsaw 02507 Poland

**Keywords:** bioprinting, granular hydrogels, microdroplets, microfluidic, tissue engineering

## Abstract

Engineering bioscaffolds with tailored architectures and optimized physicochemical properties remains a crucial yet challenging goal in tissue engineering and regenerative medicine. In this study, the design of reinforced concrete‐inspired annealed granular hydrogel (GH) scaffolds that meet the essential yet often conflicting requirements for bioscaffolds: providing adequate mechanical strength while facilitating cell infiltration, nutrient exchange, and the formation of complex cellular networks. GH building blocks are synthesized using a binary macromonomer system of hyperbranched polyethylene glycol and thiolated gelatin within microfluidic droplets, benefiting from the molecular interface assembly and templating effects of the microdroplets, which possess highly reactive vinyl functional groups, thereby endowing the annealed GH scaffolds with highly customizable properties. The versatility of this platform is demonstrated by the creation of full‐thickness engineered skin tissues that support keratinocyte attachment and differentiation; the formation of a mature epidermis, complete with a developed stratum corneum, and the expression of key markers, such as keratin 10 and keratin 14, while minimizing contraction over long‐term culturing, a common limitation of traditional collagen‐based scaffolds. Owing to their biocompatibility, tunable mechanical properties, ease of surface functionalization, and compatibility with bioprinting, these scaffolds have significant potential for applications in tissue engineering, drug delivery, and bioprinting.

## Graphical abstract

This work introduces a highly customizable granular hydrogel scaffold, designed from reactive microparticles through binary macromonomer microdroplet assembly, achieving a precise balance of mechanical strength and biological functionality.

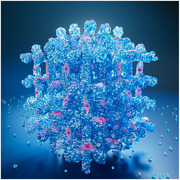

## 1 Introduction

Skin tissue engineering involves isolating and culturing skin cells, preparing biocompatible scaffolds, seeding cells, and maturing them using growth factors to restore the structure and function of damaged skin.^[^ ^1^, ^2^, ^3^, ^4^, ^5^ ^]^ These processes are critical for reestablishing the protective barrier of the skin, preventing water loss, and regulating temperature. Additionally, they enable the development of ex vivo models that closely mimic human skin, supporting the study of disease mechanisms and drug screening.^[^ ^6^, ^7^, ^8^ ^]^ Central to these advancements are scaffolds that provide essential support for cell attachment, migration, growth, and differentiation, thus enabling the formation of complex skin tissues. Collagen‐based hydrogels are widely used in this field owing to their biocompatibility and cell‐binding properties. However, they face limitations such as weak mechanical strength, rapid degradation, immunogenicity, and shrinkage during fibroblast culturing, which affect the quality of the engineered skin.^[^ ^9^, ^10^ ^]^ To overcome these issues, crosslinking strategies and composite systems have been introduced to enhance the mechanical properties and stability of collagen hydrogels.^[^ ^11^, ^12^, ^13^, ^14^ ^]^ Additionally, non‐extracellular matrix (ECM) natural and synthetic polymers have been explored as alternative scaffolds.^[^ ^15^, ^16^, ^17^ ^]^ However, these approaches often involve cytotoxic crosslinking agents, lack bioactive sites, and require complex fabrication processes, which increase preparation time and labor.

Granular hydrogels (GHs) composed of packed hydrogel microparticles (HMPs) have recently gained significant attention in various fields, such as tissue engineering, drug delivery, and bioprinting.^[^ ^18^, ^19^, ^20^, ^21^, ^22^ ^]^ These hydrogels offer high porosity and permeability, which promote cell migration and facilitate nutrient and waste exchange, as well as injectability for minimally invasive applications and tunable mechanical properties through adjustments in the HMP size, composition, and crosslinking density. Studies have also shown that the immune response to GHs in vivo differs from the response to conventional bulk hydrogels.^[^ ^23^, ^24^, ^25^, ^26^ ^]^ However, traditional GHs, which rely on physical jamming effects, often lack self‐supporting structures owing to weak interparticle forces, limiting their direct use in tissue scaffolds. To address this limitation, two strategies have been developed: bioprinting within a gel bath^[^ ^27^, ^28^, ^29^ ^]^ and employing physical or chemical methods to strengthen the HMP interactions.^[^ ^30^, ^31^, ^32^, ^33^ ^]^ Although bioprinting within a gel bath allows for high‐resolution scaffold creation, scaffolds must still undergo postprinting cultivation, requiring integration with bonding techniques to establish durable structures. Thus, customizing HMP properties, particularly the surface chemistry, is essential to enhance the annealing processes and further modify their mechanical and chemical characteristics, thereby optimizing the cellular microenvironment. For example, Segura and co‐workers pioneered the use of transglutaminase to catalyze covalent bonding between peptide residues on HMPs,^[^ ^34^ ^]^ whereas Burdick and Anseth introduced macromolecule precursors with reactive functionalities to tailor the chemical reactivity.^[^ ^35^, ^36^, ^37^, ^38^, ^39^, ^40^ ^]^ Despite these advances, many methods involve complex, nongeneralizable crosslinking processes, posing challenges to their widespread application.

**Figure 1:**
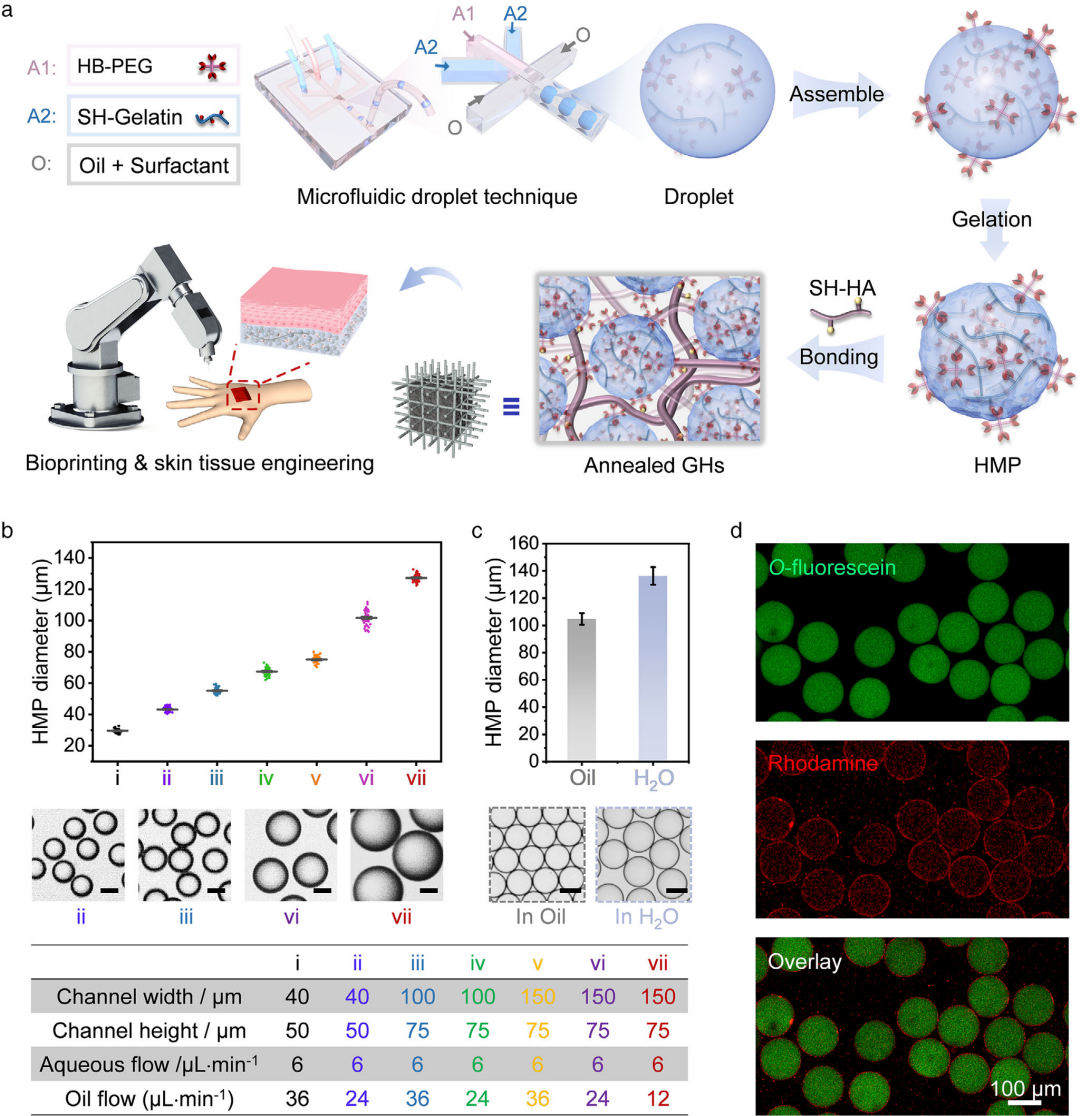
a) Schematic diagram depicting the complete construction process of reinforced concrete‐inspired annealed GHs as tissue engineering scaffolds. b) Influence of microfluidic channel dimensions and fluid flow rate on the diameter of the generated microdroplets. c) Swelling behavior of the prepared HMPs after transfer from the oil phase to PBS buffer. d) Confocal images of *O*‐fluorescein‐labeled HMP aggregates after coculturing with SH‐HA‐Rhodamine.

Alkene chemistry is a versatile tool in organic synthesis and is central to reactions such as hydrogenation, halogenation, hydration, polymerization, and Michael addition.^[^ ^41^, ^42^, ^43^, ^44^ ^]^ Introducing alkene functional groups onto HMP surfaces offers advantages such as simplified surface functionalization and effective crosslinking via established chemical processes. However, the practical use of vinyl groups in HMPs is limited by challenges such as alkene monomer consumption; overuse of multivinyl crosslinkers, leading to excessively rigid hydrogels; and low reactivity of long‐chain alkenes, which often require harsh conditions for further reactions. The development of efficient methods for preparing reactive alkene‐functionalized HMPs remains an important challenge in this field. In this study, we designed reinforced concrete‐inspired annealed GHs as tissue engineering scaffolds, which successfully reconcile the seemingly contradictory requirements for bioscaffolds: providing adequate mechanical strength while facilitating cell infiltration, nutrient exchange, and the formation of complex cellular networks. The building blocks of these GHs, HMPs, were synthesized using a binary macromonomer system composed of hyperbranched polyethylene glycol (HB‐PEG) and thiolated gelatin (SH‐gelatin) within microfluidic droplets (**Figure** **1**; Figures S1 and S2, Supporting Information). Owing to the intrinsic surface activity of HB‐PEGs, they spontaneously accumulate at the water‐in‐oil (W/O) droplet interface during gelation, resulting in the formation of crosslinked HMPs with enriched surface acrylate groups. These electron‐deficient acrylate groups are highly reactive, allowing HMPs to be easily functionalized with various bioactive molecules, such as RGD peptides, via alkene chemistry, thus enhancing cellular interactions. Moreover, by incorporating thiolated hyaluronic acid (SH‐HA), these HMPs can be annealed to form GH scaffolds with improved mechanical strength. In this annealed structure, the HMPs act as reinforcing elements, akin to rebars in concrete, while SH‐HA serves as the binding matrix, linking the HMPs through Michael addition reactions between its thiol groups and the vinyl functional groups on the HMP surface. This combination not only provides structural support but also creates a favorable environment for cell attachment, proliferation, and migration, while preventing shrinkage caused by cell traction‐mediated contraction. As a proof‐of‐concept, these scaffolds enable the construction of full‐skin substitutes with enhanced structural integrity. This novel material system, compatible with 3D bioprinting, is highly customizable, mimics the ECM, and has significant potential in advanced tissue engineering applications.

## 2 Results and Discussions

In contrast to traditional hydrogel preparations using small‐molecule monomer precursors, a binary system of HB‐PEG and SH‐gelatin macromonomers was used to create HMPs. This approach facilitates a simpler formulation and milder gelation process via the thiol–ene reaction, providing numerous advantages, including increased precursor reactivity, enhanced biocompatibility, and reduced cytotoxicity.^[^ ^45^, ^46^ ^]^ As illustrated in Figure 1a, HMPs were synthesized by crosslinking SH‐gelatin with HB‐PEG within uniform microfluidic droplets using a W/O segmentation technique.^[^ ^31^, ^47^ ^]^ In this method, aqueous solutions of SH‐gelatin and HB‐PEG were introduced separately into the microfluidic droplet device as the discontinuous phase, while methyl silicone oil with 5 wt% XIAMETER RSN‐0749 resin surfactant served as the continuous phase. The SH‐gelatin and HB‐PEG aqueous solutions were intentionally kept separate until they met within the microchannels, where they were immediately sheared into droplets by the oil phase and thoroughly mixed via enhanced collision‐induced mixing between the droplets and microchannels. This setup ensured thorough mixing of the macromonomer precursors and prevented premature crosslinking of SH‐HA and HB‐PEG, thereby reducing the risk of blockage in the microchannels. After collection, the microdroplets were transferred to a Petri dish and reacted at room temperature for 1 h to achieve full gelation. By adjusting the flow rate ratio of the two phases or altering the microchannel dimensions (Figure 1b), uniform HMPs of varying sizes were produced with a coefficient of variation (CV) of less than 5%. For subsequent biomedical applications, a mixture of hexane and ethyl acetate was used to efficiently remove the residual oil and surfactant, followed by redispersion of the HMPs in phosphate‐buffered saline (PBS) solution. Upon redispersion in PBS, the size of the HMPs increased slightly owing to water absorption (Figure 1c).

Coculturing HMPs with Rhodamine‐labeled SH‐HA (200–400 kDa) resulted in the emergence of red fluorescence on their surfaces (Figure 1d). This observation indicates that the gelation process, which is influenced by the uneven distribution of macromolecular reactive sites and steric hindrance, did not fully consume the acrylate functional groups of HB‐PEG in a stoichiometrically balanced manner.^[^ ^33^, ^38^ ^]^ As a result, the HMPs retained a certain number of reactive acrylate groups. Owing to the high molecular weight of Rhodamine‐labeled SH‐HA, its penetration into the HMPs was restricted, causing it to react with the active acrylate groups mainly at the HMP surface. This led to red fluorescence being confined to the outer surface of the HMPs, highlighting their favorable surface reactivity.

**Figure 2:**
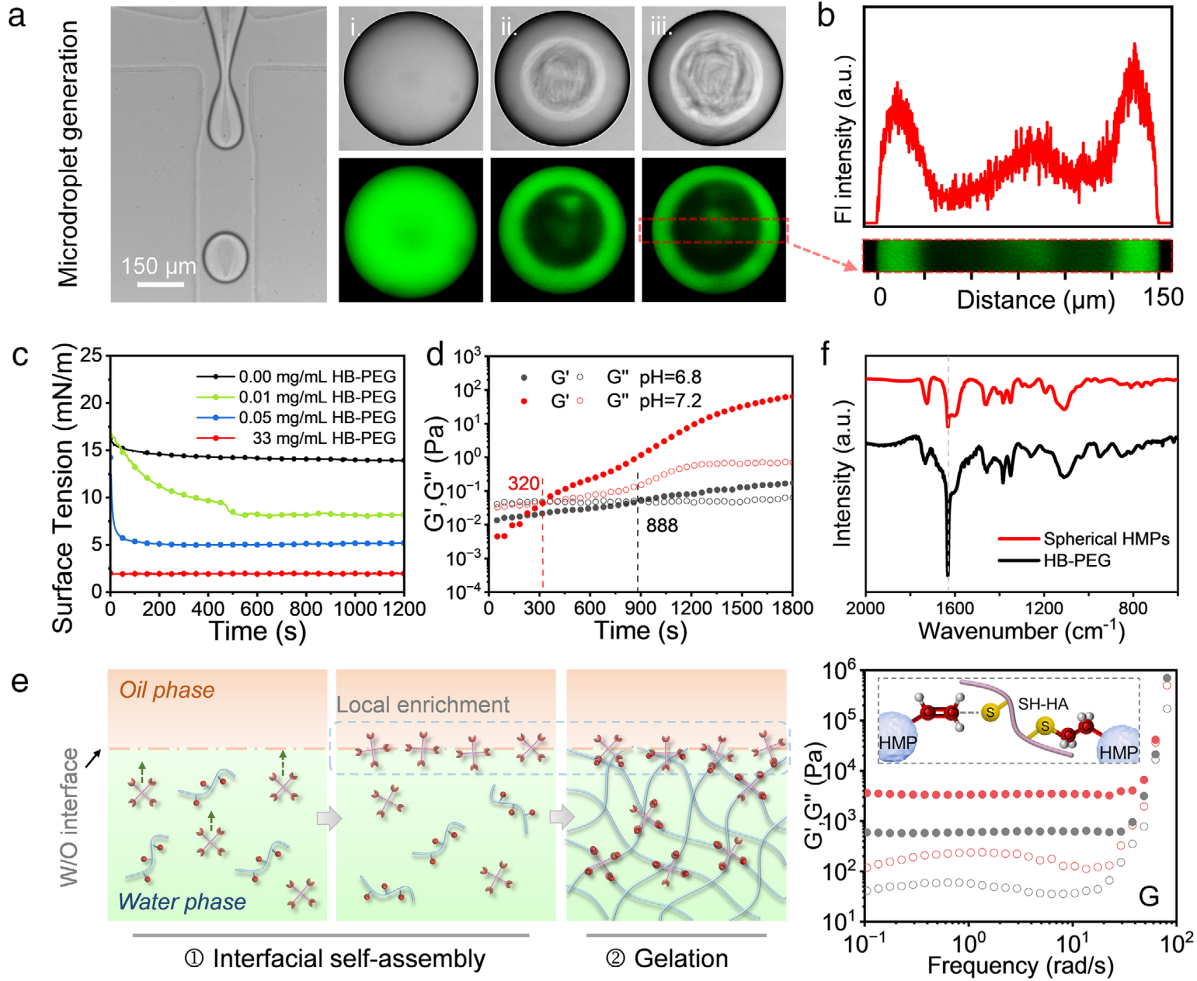
a) Temporal evolution of droplet morphology and the distribution of *O*‐fluorescein‐labeled HB‐PEG after droplet formation. Images i, ii, and iii show the microdroplets immediately after formation, at 15 s, and at 5 min, along with corresponding confocal images. b) Fluorescence intensity distribution along the central axis in HMPs. c) Changes in surface tension over time in water‐in‐oil (W/O) droplets containing HB‐PEG at different concentrations. d) Time sweep rheology experiments on hydrogel samples prepared under varying pH conditions. e) Schematic representation of the mechanism for producing highly reactive HMPs with acrylate‐rich surfaces through initial interfacial assembly of HB‐PEG, followed by gelation. f) FT‐IR spectra comparing HB‐PEG and synthesized HMPs. g) Crosslinking of HMPs with SH‐HA, forming annealed GHs (inset), with frequency sweep tests conducted before (gray dashed line) and after annealing (red dashed line).

To determine the mechanisms driving the surface reactivity, the assembly and gelation processes of the HMPs within the microdroplet templates were thoroughly examined. *O*‐fluorescein, which emits green fluorescence under blue or UV light, was used to label the HB‐PEG macromonomers, allowing precise tracking of HB‐PEG's temporal and spatial distribution of HB‐PEG during gelation. As illustrated in **Figure** **2**a, the microdroplets uniformly fluoresced when they initially formed in the microchannel (Figure 2a(i)), indicating a homogeneous distribution of HB‐PEG. However, ≈15 s after formation, a dynamic liquid film began to form at the exterior of the droplet (Figure 2a(ii)), with concentrated green fluorescence near the surface. After 5 min (Figure 2a(iii)), the liquid film expanded further, whereas the fluorescence intensity measurements along the central axis showed significantly higher fluorescence at the edges than at the center (Figure 2b), indicating a localized concentration of HB‐PEG at the water–oil interface. To understand the uneven distribution of HB‐PEG, the dynamic surface tension of the W/O microdroplets was analyzed. As shown in Figure 2c, compared with microdroplets composed of pure water, the presence of HB‐PEG significantly reduced the interfacial tension of the droplets. Moreover, as the concentration of HB‐PEG increased, the time required for the interfacial tension to reach its minimum decreased from ≈500 s to only a few seconds—much shorter than the gelation time, which spans several minutes (Figure 2d). This finding indicates that HB‐PEG has strong interfacial activity, enabling its rapid accumulation at the droplet surface prior to the gelation of the hydrogel precursors. This localized enrichment of HB‐PEG at the microdroplet interface within the confined environment resulted in a higher concentration of HB‐PEG than SH‐gelatin during the crosslinking process (Figure 2e). This imbalance led to a surplus of acrylate groups from HB‐PEG relative to thiolate groups from SH‐gelatin, leaving a substantial number of unreacted reactive acrylate groups on HMP surface. The Fourier‐transform infrared spectra also supported this reasoning, revealing an absorption peak at ≈1640 cm^−^¹, indicative of C═C double bonds in the resulting HMPs (Figure 2f). Consequently, a simple and efficient one‐step method was developed to synthesize HMPs with surfaces enriched with reactive acrylate groups.

**Figure 3:**
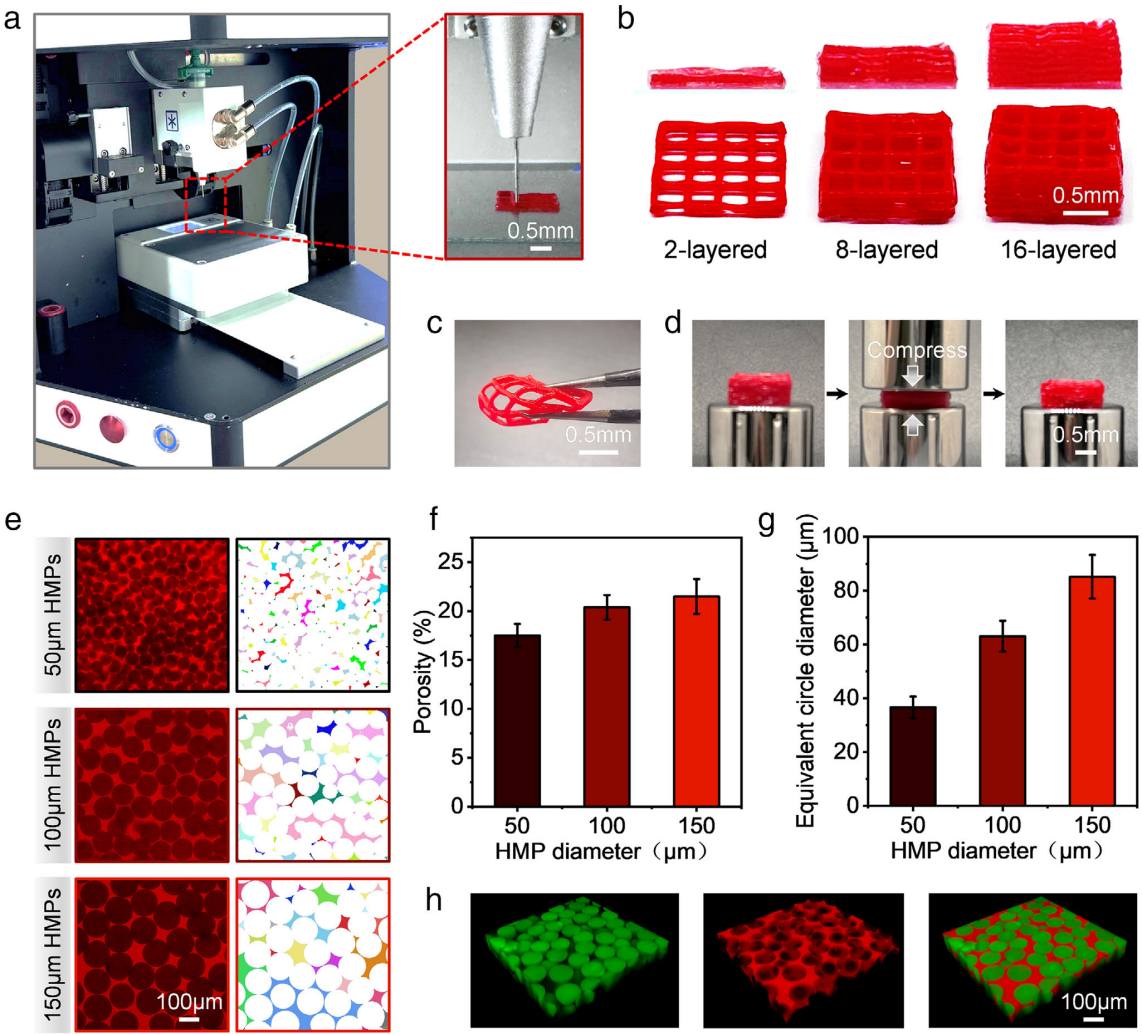
a) Bioprinting experimental setup utilizing densely packed HMPs and SH‐HA as a dual‐component bioink. The inset within the red box on the right displays a close‐up of the extruded filament from a 21G conical nozzle. b) Progression of printed structures with varying thicknesses using the dual‐component bioinks. c) The printed structures show excellent flexibility, maintaining their structural integrity even when bent with tweezers. d) The structures also exhibit strong resistance to compression. e) Porosity of the GHs quantified from 2D slices of confocal stacks, along with f) calculated porosity values and g) equivalent circular diameters of the pores. h) 3D reconstruction reveals interconnected macropores in the annealed GHs.

This advancement not only enables precise customization of the surface physicochemical properties of HMPs through versatile alkene chemistry but also significantly enhances their mechanical properties through interparticle crosslinking when used as scaffold materials. As illustrated in Figure 2g, the incorporation SH‐HA (Figure S3, Supporting Information) into the densely packed HMP aggregates, which formed annealed GHs, substantially increased the mechanical strength of the resultant structure. In comparison to GHs formed from densely packed HMPs whose surface acrylate groups had been fully consumed by excess Rhodamine B‐PEG‐thiol (RB‐PEG(20k)‐SH), the modulus increased from 0.6 to 3.4 kPa, representing a nearly sixfold enhancement. This notable enhancement indicates that SH‐HA acts as an adhesive, linking the HMPs via a Michael addition reaction between SH‐HA's thiol groups and the acrylate groups on the HMP surfaces. Analogous to reinforced concrete, HMPs serve as reinforcing components akin to rebar, whereas SH‐HA functions as the binding matrix, effectively connecting the HMPs and forming annealed GHs with superior mechanical performance. Moreover, HMPs with surfaces rich in reactive acrylate groups exhibit tunable reaction rates with SH‐HA, often referred to as “annealing” rates, which can be modulated by adjusting the pH.^[^ ^38^, ^43^ ^]^ This tunability ensures the system's injectability and allows for postinjection curing and shaping. Consequently, HMPs with reactive acrylate groups combined with SH‐HA offer a promising new class of bioinks. As shown in **Figure** **3**a, this bioink, consisting of densely packed HMPs and SH‐HA, can be easily extruded through a 21G nozzle using a standard extrusion‐based bioprinter, producing GH filaments that enable the creation of grid‐shaped prints with a thickness of over 16 layers (Figure 3b). By contrast, printing with densely packed HMPs alone, which primarily relied on physical jamming for cohesion, resulted in weaker structures that lacked sufficient structural stability and were prone to collapse (Figure S4, Supporting Information). Furthermore, the prints formed via interparticle crosslinking exhibited exceptional self‐supporting properties, allowing them to be lifted and manipulated with forceps while maintaining their structural integrity, even when bent or compressed (Figure 3c,d). This demonstrates the substantial mechanical strength imparted by SH‐HA‐mediated annealing of GHs.

In addition to their enhanced mechanical properties, the structural characteristics of the annealed GHs, including the porosity, pore size, and pore quantity, were systematically evaluated using confocal microscopy. Image acquisition and analysis followed the methodology outlined by Burdick and co‐workers^[^ ^48^ ^]^ using ImageJ software. Specifically, to visualize the pore structures, tetramethylrhodamine isothiocyanate‐labeled dextran (average molecular weight 20 kDa) was introduced into the dispersion medium surrounding the HMPs, and confocal imaging was conducted at a frequency of 400 Hz and a resolution of 1024 × 1024 pixels, with each region of interest comprising at least 50 slices and a z‐step size between 2 and 3 µm. Pore characteristics, such as porosity, pore area, and pore number, were quantified by analyzing individual 2D slices from the confocal stack, as illustrated in Figure 3e, where distinct pores are color‐coded in the right panel. The overall porosity of each sample was calculated as the average porosity obtained from 30 adjacent slices. As summarized in Figure 3f, the data demonstrate a positive correlation between the HMP size and porosity, with a constant packing density achieved through centrifugation at 8000 rpm for 5 min. Additionally, mean pore size was determined using the equivalent circle method, showing an increase from 38 to 62 to 85 µm as HMP size increased from 50 to 100 to 150 µm (Figure 3g). Figure 3h shows 3D reconstructions of the annealed GHs, illustrating the formation of interconnected pore structures. These relatively large pore sizes, combined with the interconnected nature of the pores, indicate that the annealed GHs have significant potential as scaffolds for tissue engineering applications.

**Figure 4:**
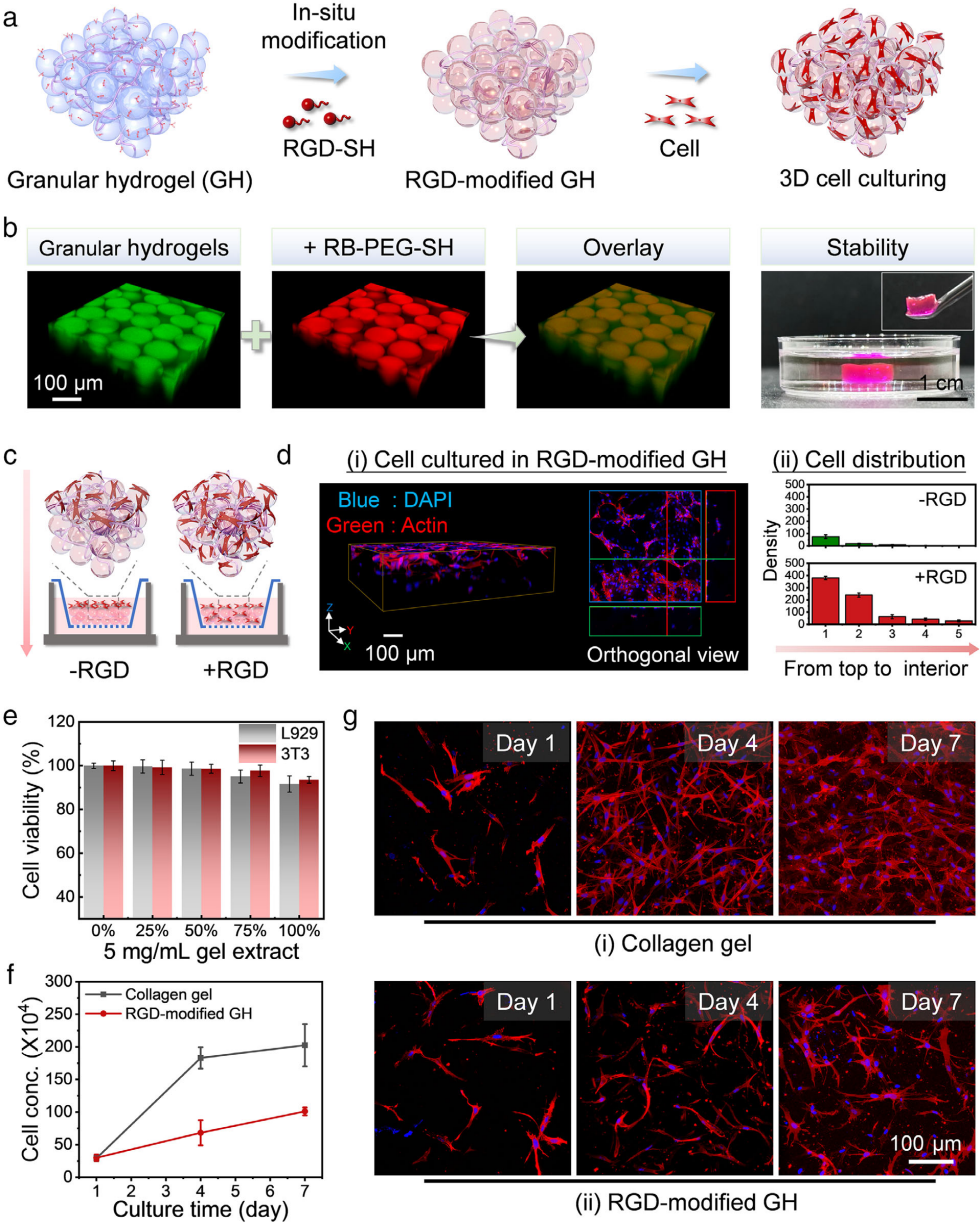
a) Schematic representation of the construction of GHs, surface modifications, and their application as scaffolds for cellular growth. b) 3D reconstruction of the annealed hydrogel following Rhodamine B‐PEG(20k)‐Thiol modification, achieved by incubating RB‐PEG(20k)‐SH with the annealed GH scaffold under neutral conditions at room temperature for 1 h. From left to right: 3D topology of *O*‐fluorescein‐labeled HMPs, distribution of grafted Rhodamine B, and an overlay of the two images. The digital image on the far right shows that the annealed GH maintains its structural integrity after immersion in PBS buffer. c) Schematic of cell migration within a transwell insert. d) Distribution of cells within the hydrogel after 3 days of culture. e) Cell viability of L929 and 3T3 cells after treatment with the hydrogel extracts. f,g) Comparison of HDF proliferation and network formation in conventional collagen hydrogels versus annealed GHs.

In addition to being compatible with additive manufacturing technologies for creating scaffolds with enhanced mechanical strength and matching pore structures, customizable surface properties are crucial for promoting cell adhesion, proliferation, and migration on scaffolds. In this context, the abundant acrylate groups in HMPs offer a significant advantage by retaining unreacted acrylate groups, preserving chemical reactivity even after annealing, and enabling convenient postfabrication modifications (**Figure** **4**a). RB‐PEG(20k)‐SH was used as a model molecule for scaffold modification. Its small molecular size allowed it to permeate the microparticle network, whereas its thiol group reacted with excess acrylate bonds. As shown in Figure 4b, after incubation with RB‐PEG(20k)‐SH, the annealed GH scaffold exhibited a strong red fluorescence uniformly distributed across the HMP building blocks, highlighting the excellent reactivity and ease of surface and internal modification of the annealed GH‐based scaffolds. Building on this, the scaffold was further modified with thiolated RGD peptides (G–R–G–D–S–P–C) using the same incubation method, resulting in RGD‐modified annealed GHs. The impact of RGD grafting on cell migration into the scaffold was assessed by seeding human dermal fibroblast cells (HDFs) onto RGD‐modified annealed GHs within a transwell insert (Figure 4c). As shown in Figure 4d, RGD‐modified scaffolds significantly enhanced cell migration into the scaffold interior, whereas the cells were unable to penetrate the unmodified scaffold. This improvement is likely due to the enhanced cell‐scaffold adhesion facilitated by RGD surface modification. Given the availability of various commercially available thiolated proteins and bioactive components, the customizable physicochemical properties of these GHs offer significant potential for their application as versatile 3D bioscaffolds in tissue engineering.

**Figure 5:**
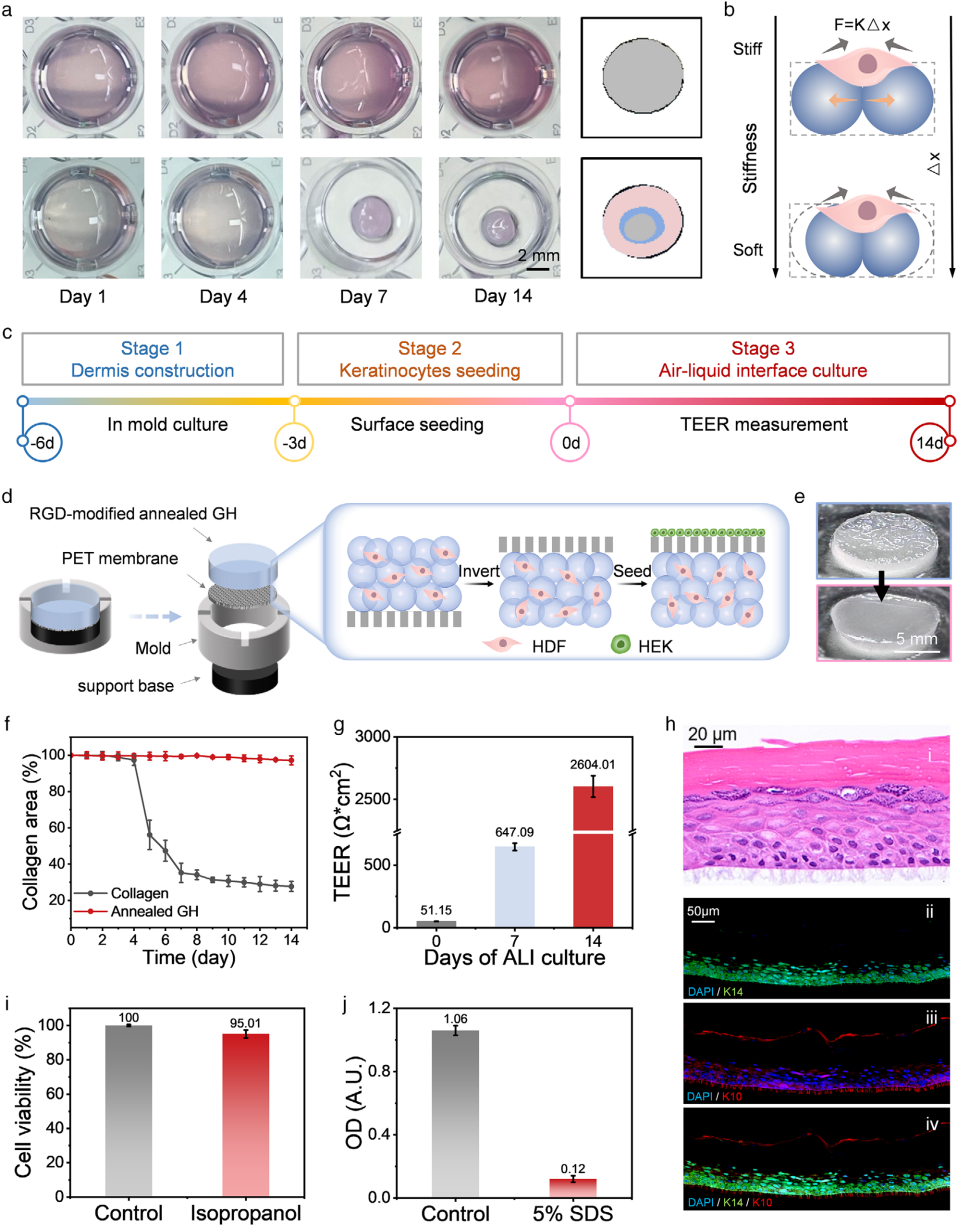
a) Measurement of the contraction in collagen hydrogels and annealed GHs with encapsulated HDFs. b) Mechanism of contraction resistance in the annealed GH. c) Experimental workflow for constructing a full‐thickness skin model using the annealed GH. d) Schematic illustration of the skin model construction in the mold. e) The PET membrane promotes the formation of a smooth surface on the annealed GH, facilitating the seeding of a monolayer of keratinocytes. f) Contraction behavior of different scaffolds during cell culture. g) Resistance measurements of the HDF‐encapsulated GH overlaid with keratinocytes during air–liquid interface culture. h) Epidermal stratification as observed through hematoxylin and eosin (H&E) staining (i), along with multiplex immunohistochemistry (mIHC) staining for keratin 10 (K10, ii), keratin 14 (K14, iii), and their corresponding overlay (iv). Cell viability was assessed by exposing the skin model to i) isopropanol and j) 5 wt% SDS, with PBS used as the control.

Although the presence of excess acrylate groups in scaffold materials may raise potential cytotoxicity concerns, it is important to note that the acrylate functionality of these GHs is derived from hyperbranched HB‐PEG macromonomers. Previous studies have demonstrated that the highly compact topology of these polymers minimizes the cytotoxic effects of the acrylate groups on cells.^[^ ^49^ ^]^ To assess the cytocompatibility of the annealed GHs, the viability of two mouse fibroblast cell lines, L929 and 3T3, was evaluated after treatment with hydrogel extracts at varying concentrations. As shown in Figure 4e, the annealed GH scaffolds exhibited excellent biocompatibility and minimal cytotoxicity under all tested conditions. In addition, the proliferation of human HDFs embedded within the GH scaffold was evaluated, with the proliferation of HDFs in collagen hydrogel—a widely recognized biocompatible material—used as a control. Figure 4f reveals that HDF cells proliferated in both scaffolds, forming intricate network structures (Figure 4g); however, proliferation and network formation occurred more rapidly in the collagen hydrogel, likely owing to its lower modulus and enhanced matrix remodeling. Despite these advantages, the rapid degradation and insufficient mechanical strength of collagen hydrogels limit their ability to maintain their structural integrity. As shown in **Figure** **5**a, the collagen hydrogel significantly contracted, shrinking to 15% of its original size during long‐term culturing owing to the traction forces exerted by the proliferating HDFs. By contrast, the annealed GH scaffolds retained their original dimensions and exhibited remarkable contraction resistance. This can be attributed to the scaffold's unique combination of seemingly contradictory structural properties. The pores within the interstitial spaces between the HMPs facilitated cell migration and proliferation, whereas the HMPs remained relatively intact under standard cell culturing conditions (Figures S5 and S6, Supporting Information). Consequently, the volumetric repulsive forces between the HMPs and the cohesive forces from cellular traction balanced each other, effectively counteracting scaffold contraction (Figure 5b). Therefore, annealed GH effectively meets these seemingly conflicting requirements by providing sufficient mechanical strength and supporting cell infiltration, nutrient exchange, and complex network formation, making it a promising solution for tissue engineering.

The annealed GH scaffold was specifically applied to construct full‐thickness skin tissue, addressing a major challenge in the field—scaffold contraction—which can lead to defects in skin models and compromise their utility in skin‐related assessments, such as irritancy testing, permeability evaluation, and drug‐retention studies.^[^ ^15^ ^]^ As depicted in Figure 5c and Figure S7 (Supporting Information), the process of constructing a full‐thickness skin involves sequential steps: first, the dermal layer is formed, followed by keratinocyte seeding on its surface. Once a complete keratinocyte layer is established, an air–liquid interface (ALI) culture is applied to promote differentiation and maturation, ultimately resulting in a fully developed, full‐thickness skin structure. HDF‐encapsulated, annealed GH was used as the dermal layer. To address the surface roughness, which can impair keratinocyte attachment and differentiation, a cylindrical mold with a porous polyethylene terephthalate (PET) membrane at the base was used. By casting the HDF‐encapsulated hydrogel into a mold and inverting it, a smooth upper surface was obtained, facilitating efficient keratinocyte seeding and subsequent development (Figure 5d,e).

Compared with the collagen control, the annealed GH scaffold significantly minimized contraction during culturing of the dermal layer (Figure 5f). Electrical resistance, an indicator of skin barrier integrity and hydration levels, was measured at the onset of ALI culturing. As shown in Figure 5g, the resistance values progressively increased from 50 to 2600 Ω, reflecting the gradual development of a robust barrier. Hematoxylin and eosin staining of sections revealed an ≈50 µm thick epidermal layer topped by a keratinized stratum corneum comparable to conventional skin equivalents, such as EpiDerm (Figure 5h,i; Figure S8, Supporting Information). Additionally, the expression of epidermal differentiation markers confirmed the successful formation of the keratinized stratum corneum, with keratin 10 expressed in the upper layers and keratin 14 in the basal layers, indicating a well‐differentiated epidermis (Figure 5h(ii–iv)). Skin irritation tests were performed according to the OECD 439 standard protocols. Given the lack of vascularization in these models, the assessment focused on evaluating the initial events, specifically the cell damage caused by chemical penetration through the stratum corneum. Hence, cell viability was used as the primary indicator of irritation. Irritant chemicals were identified by their ability to decrease cell viability below established thresholds—commonly set at ≤50%, as defined by standards such as UN GHS Category 2. By contrast, chemicals that maintained cell viability above this threshold were classified as nonirritants. The test substances, including isopropanol and 5% w/v sodium dodecyl sulfate (SDS), which are commonly used in validation studies, were applied topically to the surfaces of the skin models. As illustrated in Figure 5i,j, keratinocytes exposed to isopropanol retained 95% cell viability, indicating nonirritant properties, whereas SDS reduced cell viability to 11%,^[^ ^50^, ^51^ ^]^ indicating significant irritant potential. These outcomes are consistent with previous studies that classified isopropanol as a nonirritant and SDS as an irritant. It is important to emphasize that additional studies are necessary to evaluate the irritation potential of unclassified chemicals using this tissue model to fully meet the OECD criteria for sensitivity, specificity, and accuracy; however, this is beyond the scope of this study.

## 3 Conclusion

This study introduces a strategy for synthesizing vinyl‐functionalized HMPs using a binary macromonomer system comprising HB‐PEG and SH‐gelatin within microfluidic droplet templates. By leveraging the spontaneous enrichment of HB‐PEG at the O/W interface of the microdroplets prior to gelation, this approach enables the production of HMPs with a uniform size distribution, tunable mechanical properties, and, notably, a high density of reactive acrylate groups on their surfaces, allowing for customizable surface modifications and interparticle crosslinking. The versatility of these HMPs is exemplified by their use as bioinks for fabricating custom‐designed annealed GH scaffolds via extrusion‐based 3D printing. Following postmodification with the bioadhesive peptide RGD, the annealed GHs effectively promoted cell attachment and differentiation, leading to the formation of a full‐thickness skin model with a well‐developed stratum corneum and appropriate expression of key differentiation markers, including keratin 10 and keratin 14. In addition, these hydrogels minimally contracted during long‐term culturing, addressing the critical challenge posed by matrix contraction from cellular traction forces, a limitation commonly associated with conventional collagen‐based scaffolds. This property is particularly advantageous for the development of in vitro testing models. This study presents a versatile and scalable approach for the fabrication of HMP‐based GHs with finely tunable physicochemical properties. Combining their superior biocompatibility, mechanical durability, and customizable surface functionality, these materials have significant potential for advancing tissue engineering, drug delivery, and bioprinting applications, distinguishing them from current scaffold technologies.

## Conflict of Interest

The authors declare no conflict of interest.

## Supporting information



Supporting Information

## Data Availability

The data that support the findings of this study are available in the supplementary material of this article.
