# Supplementary material for: Reinforced Granular Hydrogels Scaffolds with Tunable Physicochemical Properties for Advanced Skin Tissue Engineering
Source: Adv Sci (Weinh). 2025 May 5;12(21):2415634. doi: 10.1002/advs.202415634 (PMC12140292; doi:10.1002/advs.202415634)
Supplement: Supplementary file 1 — Supporting Information [file ADVS-12-2415634-s001.docx]

Supporting Information for

Reinforced Granular Hydrogels Scaffolds With Tunable Physicochemical Properties for Advanced Skin Tissue Engineering

J. Zhang, Y. Wang, Y. Liu, G. Wu, G. Lu, Y. Li, Y. Shen, C. Wang, M. Khanmohammadi, W. Święszkowski, J. Wang,* Z. Yu*

J. Zhang, Y. Wang, Y. Liu, G. Wu, G. Lu, Y. Li, Y. Shen, C. Wang, Z. Yu

State Key Laboratory of Materials-Oriented Chemical Engineering, College of Chemical Engineering, Nanjing Tech University, 30 Puzhu South Road, Nanjing, 211816 P. R. China
E-mail: [ziyi.yu@njtech.edu.cn](mailto:ziyi.yu@njtech.edu.cn)

J. Wang

Reproductive Medicine Center, Zhongshan Hospital, Fudan University, Shanghai, 200032 P. R. China
E-mail: [wang.jing4@zs-hospital.sh.cn](mailto:wang.jing4@zs-hospital.sh.cn)

M. Khanmohammadi, W. Święszkowski

Faculty of Materials Science and Engineering, Warsaw University of Technology, 02507 Warsaw, Poland

**Experimental methods:**

*Synthesis of Hyperbranched Poly(ethylene Glycol) Diacrylate -Poly(propylene glycol) Diacrylate Copolymer Macromonomers (HB-PEG)*^[1]^: In a round-bottomed flask, a solution was prepared by dissolving 11.50 g (20 mmol) of polyethylene glycol diacrylate (PEGDA; average Mn ~ 575, Sigma-Aldrich) and 3.5 g (5 mmol) of poly(propylene glycol) diacrylate (PPGDA; average Mn ~ 700, Adamas-beta) in 62.5 mL of butanone (Shanghai Lingfeng Chemical Reagent Co., Ltd). The mixture was stirred until it became fully homogeneous. Subsequently, 0.23 g (1.4 mmol) of azobisisobutyronitrile (AIBN; Aladdin Shanghai Reagent Co., Ltd) and 0.30 g (1.0 mmol) of tetraethylthiuram disulfide (DS; Aladdin Shanghai Reagent Co., Ltd) were added to the mixture. The solution was purged with argon gas for 1 h to remove dissolved oxygen before initiating the reaction. The mixture was then heated to 70 °C under continuous stirring to initiate polymerization. The molecular weight and conversion of the products were monitored by removing small aliquots of the reaction mixture from the flask, diluting them with dimethylformamide (DMF; 99.9%, Shanghai Macklin Biochemical Technology Co., Ltd.), and analyzing them using gel permeation chromatography (GPC; Agilent 1260 Infinity II Multidetector GPC System). Upon reaching the desired molecular weight and conversion (typically > 15%), the reaction was terminated, air was added, and the mixture was allowed to cool to room temperature. To isolate the target product, the polymer was precipitated using a 1:2 (v/v) mixture of hexane (Shanghai Lingfeng Chemical Reagent Co.) and ether (Shanghai Lingfeng Chemical Reagent Co.), and any residual solvent was removed via rotary evaporation. Unless speciﬁed, HB-PEG samples with a molecular weight (Mw) of 11.7 kDa and a polydispersity index of 3.4 were used for the experiments in this work. ^1^H NMR spectra of the samples dissolved in deuterated water (D_2_O) were acquired using a Bruker Advance II 400 MHz spectrometer and analyzed using MestReNova processing software. To prepare fluorescently labeled HB-PEG, 5 mg (12.5 μmol) of fluorescein *O*-methylacrylate (Sigma-Aldrich Co., Ltd, Shanghai, China) was introduced as a copolymer monomer under the same reaction conditions, keeping all other parameters constant.

*Synthesis of Thiolated Gelatin (SH-gelatin)*: A 200 mL aqueous solution containing 2 g of gelatin (Type B, Bloom number: 188–250, with a carboxyl group content of ≈1 mmol/g, Shanghai Maokang Biotechnology Co., Ltd.) was prepared in a flask. Under continuous stirring, 2 g (8.4 mmol) of 3,3'-dithiobis(propionohydrazide) (DTP; Frontier Specialty Chemicals) was introduced. The mixture was stirred for 2 h until complete dissolution. Next, 1 g (5.2 mmol) of 1-ethyl-3-(3-dimethylaminopropyl) carbodiimide hydrochloride (EDC-HCl; Aladdin Shanghai Reagent Co., Ltd.) was added to activate the carboxyl groups, and the pH was adjusted to 4.75 using 1.0 M HCl (Yonghua Chemical Co., Ltd.). The reaction proceeded at room temperature for 5 h, and then it was quenched by adjusting the pH to 7.0 using 1.0 M NaOH (Aladdin Shanghai Reagent Co., Ltd.). Next, 10 g (64.8 mmol) of dithiothreitol (DTT; Aladdin Shanghai Reagent Co., Ltd), equivalent to 6–8 times the molar amount of DTP, was added to the solution. The pH of the reaction mixture was adjusted to 8.5 using 1.0 M NaOH, and the mixture was stirred at room temperature for 24 h. The reaction was then terminated by adjusting the pH to 3.5 using 1.0 M HCl. The resulting solution was dialyzed for 2 d in the dark against a dilute HCl solution (pH 3.5) containing 0.1 M NaCl (Shanghai Macklin Biochemical Co., Ltd.); the dialysis solution was changed every 12 h. This procedure was followed by dialysis for an additional 2 d using dilute HCl (pH 3.5) alone, changing the solution every 12 h. After dialysis, the suspension was centrifuged at 4500 rpm for 10 min to remove any remaining particulates. The product was then frozen at –20 °C for 12 h, followed by freeze drying to yield a white cotton-like material. The final product was sealed under argon and stored at –20 °C for long-term preservation. ^1^H NMR spectra of the products dissolved in D_2_O were acquired using a Bruker Advance II 400 MHz spectrometer and analyzed using MestReNova processing software. For thiol quantification of SH-gelatin, Ellman's reagent solution was prepared by dissolving 19.8 mg (0.05 mmol) of 5,5′-dithiobis(2-nitrobenzoic acid) (DTNB; Shanghai Macklin Biochemical Co., Ltd.) in 5 mL of 0.1 M sodium phosphate buffer (Aladdin Shanghai Reagent Co., Ltd.), prepared in house and adjusted to pH 8, containing 1 mM ethylenediaminetetraacetic acid (EDTA; Shanghai Macklin Biochemical Co., Ltd.). A series of L-cysteine (Sigma-Aldrich) standards were prepared, and SH-gelatin samples were diluted to a concentration of 1 mg/mL in 0.1 M sodium phosphate buffer containing 1 mM EDTA. To each 250 μL standard or sample, 50 μL of Ellman's reagent solution and 2.5 mL of 0.1 M sodium phosphate buffer (pH 8) containing 1 mM EDTA were added. Both the standards and samples were incubated at room temperature for 15 min and analyzed using a microplate reader (BioTek Synergy H1, Agilent) at 412 nm. Unless otherwise specified, SH-gelatin samples with a thiol content of 0.70 mmol/g were used for subsequent experiments in this study.

*Synthesis of Thiolated Hyaluronic Acid (SH-HA):* SH-HA was synthesized according to previously reported methods.^[2]^ First, 75 mg of hyaluronic acid (Mw: 200–400 kDa, Bloomage Biotech) and 120 mg of DTP (0.5 mmol) were dissolved in 10 mL of deionized water, and the pH was adjusted to 4.75 using 1.0 M HCl. Then, 100 mg of EDC-HCl (0.5 mmol) was added, and the pH was maintained at 4.75 by incrementally adding 1.0 M HCl. The reaction mixture was stirred at room temperature for 5 h before quenching by adjusting the pH to 7.0 using 1.0 M NaOH. Afterward, 0.5 g of DTT (3.25 mmol) was introduced, and the pH was increased to 8.5 using 1.0 M NaOH. The reaction was allowed to proceed for 24 h. Upon completion, the mixture was dialyzed to remove unreacted small molecules, centrifuged to remove particulates, and lyophilized to yield a white fluffy product. The chemical structure of SH-HA was confirmed using ¹H NMR spectroscopy, and the thiolation degree was quantified using the Ellman test, as previously described. Unless specified, SH-HA samples with a thiol content of 1.07 mmol·g⁻¹ were used in subsequent experiments.

*Dynamic interfacial tension (IFT) test:* IFT was measured using the pendant-drop method and a digital tensiometer (Kruss DSA25S, Germany), which determines the surface tension by fitting the shape of the droplet to the Laplace equation and balancing the interfacial tension and gravity. In this study, the adsorption of HB-PEG at the interface induced changes in the droplet shape over time, allowing the surface tension to be tracked as a function of time. Gauge-19 medical blunt-tipped cannulas (nominal outer diameter: 1.052 mm; Shanghai Jinrong Chemical Technology Co., Ltd., China) were used to form water droplets at room temperature. For the IFT measurements, methyl silicone oil containing 5 wt% XIAMETER RSN-0749 resin surfactant was used as the oil phase, and water droplets containing various concentrations of HB-PEG or SH-gelatin were suspended in the oil phase. The IFT was measured every 1 s, until the surface tension reached equilibrium. To prevent droplet evaporation and concentration changes, measurements were conducted in a controlled humidity chamber, where the relative humidity (RH) was maintained at solution equilibrium. Each measurement was repeated at least three times to reduce experimental errors.

*Synthesis of HMPs via Microfluidic Emulsion Technique:* Polydimethylsiloxane (PDMS) microfluidic droplet-generation chips were fabricated using soft lithography, as previously described.^[3]^ The devices included fluid-focusing regions with dimensions of 40 × 40 μm, 100 × 100 μm, and 150 × 150 μm (length × width), and channel depths of either 50 or 75 μm. To synthesize HMPs, water-in-oil (W/O) droplets containing HB-PEG and SH-gelatin were created by shearing the aqueous phase with the oil phase within the chip microchannels. These droplets functioned as "microreactors" for gelation, forming HMPs. Specifically, the aqueous phase consisted of a 1:2 volume mixture of two components: (i) 10% w/v HB-PEG in PBS (pH 7.4, Beyotime, China) and (ii) 4.5% w/v SH-gelatin in PBS, with the pH adjusted to 7.4 using 1 M NaOH. All solutions were purified and sterilized via filtration through a 0.22 μm syringe filter (PES membrane, Millipore Express). Droplets were generated at room temperature using an inverted microscope stage (IX71, Olympus) equipped with a high-speed camera (Phantom Miro C110, Vision Research) for real-time monitoring. The aqueous phase was introduced into the microfluidic device in three segments: (i) the HB-PEG solution in the middle flanked by (ii) SH-gelatin solutions on either side. Each solution was loaded into separate 1 mL syringes and connected to a microfluidic chip. The oil phase, consisting of methyl silicone oil (PMX-200, Aladdin, China) with 5 wt% XIAMETER RSN-0749 resin surfactant (Dow Chemical, USA), was also loaded into a 1 mL syringe and connected to the chip. All the syringes fitted with needles were mounted on syringe pumps with polyethylene tubing, and the tubing ends were inserted into the respective PDMS microfluidic chip inlets. To generate microdroplets, the silicone oil was initially pumped at 20 μL/min to fill the channels, followed by the aqueous discontinuous phase at a combined rate of 6 μL/min (2 μL/min for each aqueous segment). The three aqueous segments remained unmixed until the droplets segmented. Monodisperse microdroplets formed as the oil phase sheared the aqueous phase. The droplet size was controlled by adjusting the flowrate ratio between the continuous and discontinuous phases, or by selecting PDMS microfluidic chips with different microchannel dimensions. The collected droplets were transferred to a Petri dish and left at room temperature for 1 h to allow sufficient time for HMP polymerization. After the synthesis, any residual oil and surfactant were removed using a 3:1 (v/v) mixture of hexane and ethyl acetate (Shanghai Lingfeng Chemical Reagent Co., Ltd., China). To prepare *O*-fluorescein-labeled HMPs, the same method was followed by substituting fluorescein-labeled HB-PEG for HB-PEG. To obtain CY5-labeled HMPs, CY5-PEG2K-SH (1 mg/mL; Xi’an Ruixi Biotech, China) was added to the HMP precursor solution.

*Rheological Test:* The time sweep of all samples was evaluated at 25 °C using an oscillatory rheometer (HAAKE RheoStress 1; Thermo Scientific, USA). The test geometry consisted of a 35 mm diameter plate. A total of 500 μL of the hydrogel precursor solution, composed of 3.33% w/v HB-PEG and 3% w/v SH-gelatin in PBS, was mixed and adjusted to the specified pH before being dispensed onto the preheated rheometer plate. Once completed, the test geometry was lowered to the desired gap height (0.5 mm) to initiate scanning at a frequency of 1 Hz and a strain of 1%. Each hydrogel sample was tested once, and each test was conducted in triplicate. The data represent the average of three tests and the corresponding standard deviation.

*Fourier Transform Infrared (FT-IR) and* *Raman Spectroscopy Measurements:* FT-IR spectroscopy was used to characterize the HB-PEG and HMP samples across a wavenumber range of 4000 to 400 cm⁻¹, with a resolution of 2 cm⁻¹ for 64 scans. The KBr pellet method was applied: for liquid HB-PEG, the ethanol-diluted sample was placed on a pure KBr tablet, whereas for HMPs, samples were mixed with KBr at a 1:9 mass ratio, ground, and then pressed into pellets for testing. Samples were characterized using a Nicolet iS10 FT-IR spectrophotometer (Thermo Fisher Scientific Ltd., USA). The FT-IR spectra were normalized, and key vibration bands were assigned to the corresponding chemical groups to verify their structural features. Raman spectra were recorded at room temperature using a confocal Raman spectrometer (LabRAM HR Evolution, Horiba, Japan) equipped with a 532 nm laser operating at a controlled power of 10 mW. A grating with 2400 grooves/mm was selected for the analysis, and the integration time was set to 25 s.

*Preparation of Granular Hydrogel (GH) and SH-HA Composite for Bioprinting:* Close-packed HMP aggregates, referred to as GH, were prepared by centrifuging the HMP suspension at 8000 rpm for 5 min, followed by careful removal of the supernatant. Subsequently, 5 mL of GH was combined with 10 mL of a 2.5% w/v solution of thiolated hyaluronic acid (SH-HA), and the pH was adjusted to ≈7.0. The mixture was centrifuged at 8000 rpm for 5 min, and the supernatant was meticulously removed. The resulting GH/SH-HA composite was gently homogenized using a pipette tip to achieve uniform consistency. This composite was then loaded into a 10 mL pneumatic syringe of a commercial 3D bioprinter (EFL-BP-6602 Pro, Yongqinquan Intelligent Equipment Co., Ltd., China) and extruded through a blunt 21 G needle under a pressure of 22 psi. The printing parameters were set to a printing speed of 600 mm/min, travel speed of 800 mm/min, and layer height of 0.4 mm. To observe the 3D structure of the prints using confocal microscopy, *O*-fluorescein-labeled HMPs were utilized to generate GH. Additionally, 0.1% w/v tetramethylrhodamine isothiocyanate-dextran (TRITC-dextran; 20 kDa, Sigma-Aldrich) was added to the GH dispersion solution. The resulting prints were placed in a glass-bottomed cell culturing dish (catalog 801001, NEST Biotechnology Co., Ltd., China) and observed using a Leica STELLARIS 5 confocal microscope. The obtained images were analyzed using LASX software, and a 3D structure with a depth of 150 μm (z-direction) was reconstructed from 68 optical slices in the x–y plane, resulting in a total volume of 581.25 × 581.25 × 150 μm^3^.

*In Vitro Degradation Study*: The degradation behavior of annealed GH was assessed by measuring its wet weight over time under different environmental conditions. Hydrogel samples (initial mass, W_0_) were incubated at 37 °C in a thermostatic shaker set to 100 rpm in the following solutions: PBS, PBS supplemented with 5 U/mL collagenase (Shanghai Acmec Biochemical Technology Co., Ltd., China) and 200 U/mL hyaluronidase (Shanghai Aladdin Biochemical Technology Co., Ltd., China), and 50 mM hydrogen peroxide solution (Sinopharm Chemical Reagent Co., Ltd., China). The incubation solutions were replaced daily. At predetermined time points, the hydrogel samples were retrieved, gently blotted to remove excess liquid, and weighed (W_t_). The degradation profiles were determined by calculating the mass retention ratio W_t_/W_0_.

*In Vivo Degradation Study*: All animal experiments were conducted in accordance with the guidelines approved by the Zhongshan Hospital Animal Management and Ethics Committee (2023-174). Male BALB/C mice (6–7 weeks old) were obtained from the Laboratory Animal Center of Fudan University and housed in a controlled environment at 22 ± 4 °C and 50–60% RH with a 12-hour light/dark cycle. The animals were provided with food and water ad libitum and acclimated for 1 week prior to the experiment. Under general anesthesia and sterile conditions, 100 μL of the CY5-labeled GH/SH-HA composite hydrogel was subcutaneously injected into the epilated dorsal region. In vivo degradation of the hydrogel was monitored using a compact preclinical in vivo fluorescent and bioluminescent imaging and analysis system (VISQUE InVivo Smart-LF, Korea), and the hydrogel degradation kinetics in the physiological environment were assessed by tracking the fluorescence intensity changes over time.

*Cell Culture:* Unless otherwise specified, all cells, culturing media, antibiotics, and sera were obtained from ProCell Life Science & Technology Co., Ltd., China. The L929 cell line was cultured in a complete growth medium consisting of minimum essential medium (MEM) supplemented with 10% fetal bovine serum (FBS) and 1% penicillin–streptomycin. 3T3 cells were cultured in Dulbecco’s modified Eagle’s medium (DMEM) supplemented with 10% FBS and 1% penicillin–streptomycin solution. Human dermal fibroblasts (HDFs) and keratinocytes (HEKs) were cultured in FbGrowth and KcGrowth media, respectively, and both cell types and their corresponding media were sourced from Guangdong Biocell Biotechnology Co., Ltd., China. Passages 4–6 of the HDFs and HEKs were used to construct a 3D full-thickness skin model. All cells were maintained in a standard cell culturing environment set at 37 °C with 5% CO₂ in a humidified incubator.

*Cytotoxicity Test.* Following the ISO 10993-5 guidelines, an MTT assay was conducted to assess sample cytotoxicity. To prepare extraction media, 10 mg of the freeze-dried GH/SH-HA composite hydrogel print was immersed in 2 mL of complete growth medium and incubated at 37 °C for 24 h. After incubation, the samples were centrifuged at 1000 rpm for 8 min. The supernatant was collected and sterilized by filtration through a 0.22 µm filter (PES, Millex) to obtain the extraction medium. The undiluted extraction medium was designated as 100% concentration. To assess the concentration-dependent effects, additional dilutions were prepared by diluting the extraction medium with complete growth medium to final concentrations of 75%, 50%, and 25% (v/v). Fresh complete growth medium (0%) was used as the negative control. L929 or 3T3 cells were seeded in 96-well plates at a density of 1 × 10^4^ cells/well and incubated at 37 °C in a humidified atmosphere containing 5% CO₂. After 24 h, the culture medium was replaced with the respective extraction medium, and the cells were incubated for an additional 24 h. MTT solution (10 µL, 5 mg/mL) from the MTT Cell Proliferation and Cytotoxicity Assay Kit (Beyotime Biotechnology) was added to each well. After incubating for 4 h at 37 °C, 100 µL of solubilization solution was added to each well, mixed thoroughly, and incubated for another 4 h at 37 °C in the cell culture incubator. The absorbance was measured at 570 nm using a microplate reader (BioTek Synergy H1 Multimode Reader), and cell viability was calculated as a percentage relative to the control group. Each test condition was performed in quintuplicate, with standard deviations shown as error bars indicating the variability in cell viability measurements.

*Cell Culture on the top surface of annealed GH*: A total of 100 μL of the GH/SH-HA composite, previously used as bioprinting ink, was transferred into a 24-well hanging insert with a translucent PET membrane at the base, featuring a 3.0 μm pore size (Guangzhou Jet Bio-Filtration Co., Ltd., China). The composite was allowed to gel fully at room temperature for 1 h to form the annealed GH. After gelation, the insert was immersed in a solution containing 1.5 mg/mL heptapeptide (sequence: G-R-G-D-S-P-C, Nanjing Impact BioScience Ltd.), with the pH adjusted to neutral or slightly alkaline, and incubated for 1 h. An annealed-GH control group that was not incubated with the heptapeptide solution was also established. After incubation, the insert containing the annealed GH was rinsed thrice with complete growth medium, with each rinse lasting 10 min. The inserts were then placed in 24-well plates. A total of 4 × 10^4^ HDF cells were seeded onto the top surface of the annealed GH. Simultaneously, 0.8 mL of the complete culture medium was added to the lower compartment of the insert. The culture medium in the lower compartment was replaced daily, and the culture was maintained for 7 d. To visualize cell migration at 1, 4, and 7 d post-seeding, the culture medium in the lower compartment was aspirated, followed by three washes with 1 mL of pre-warmed PBS, each lasting 3 min. The insert was then immersed in 1 mL of 4% paraformaldehyde solution in PBS (Beyotime Biotechnology) and fixed at room temperature for 30 min. The fixed insert was then washed by immersion in 1 mL PBS for 3 min, which was repeated three times. Subsequently, the insert was treated with 1 mL of PBS containing 0.5% Triton-X (Beyotime Biotechnology, China) at room temperature for 10 min to permeabilize the cells. After three additional PBS washes, the insert was immersed in 1 mL of YF594-Phalloidin solution (2 µg/mL in PBS, Uelandy, China) in the lower compartment and incubated at room temperature for 20 min. Following three PBS washes, the insert was transferred to 1 mL of 4',6-diamidino-2-phenylindole (DAPI) staining solution (Uelandy, China) at room temperature for 15 min and then washed with PBS. The translucent PET membrane of the insert was carefully removed using tweezers, and the annealed GH was removed, flipped upside down, and placed in a glass-bottomed cell culturing dish (catalog: 801001, NEST Biotechnology Co., Ltd., China). Image stacks were captured at various depths from the surface of the annealed GH, where the cells were initially seeded, using a Leica STELLARIS 5 confocal microscope. The acquired image data were thoroughly analyzed using LASX software, and a 3D structure with a depth of 150 μm (z-direction) was reconstructed from 220 optical slices in the x–y plane, resulting in a total volume of 581.25 × 581.25 × 150 μm^3^. Every 44 slices were compressed into a maximum intensity projection (MIP) image, and the number of cells in each MIP image was calculated using ImageJ software to determine the distribution of HDF from the surface of the annealed GH to its interior.

*Cell Culture in Annealed GH*: A total of 1.5 mL of GH was immersed in 3 mL of a solution containing 1.5 mg/mL heptapeptide (sequence: G-R-G-D-S-P-C), with the pH adjusted to neutral or slightly alkaline, and incubated at 37 °C for 1 h. The mixture was then centrifuged at 8000 rpm for 5 min, and the supernatant was removed to obtain RGD-modified GH. Subsequently, 350 μL of the RGD-modified GH was combined with 700 μL of a 2.5% w/v SH-HA solution, and the pH was adjusted to ≈7.0. The mixture was centrifuged again at 8000 rpm for 5 min, and the supernatant was meticulously removed before adding 25 μL of a cell suspension (4 × 10⁶ cells/mL). The resulting GH/SH-HA/HDF composite was gently homogenized using a pipette tip to achieve uniform consistency. This mixture was then added to a 48-well plate and incubated at 37 °C for 30 min before adding 500 μL of FbGrowth medium. To observe cell growth within the annealed GH at 1, 4, and 7 d, the same fixation and staining methods described previously were employed. For the control, HDF cells were cultured in collagen hydrogel, prepared by mixing 1.1 mL of collagen type I solution (rat tail, Sigma-Aldrich), 200 μL of 10X PBS, and 600 μL of FbGrowth medium in an ice bath. The pH was neutralized using 100 μL of 0.1 M NaOH to form a collagen pre-gel solution. This was mixed with 1.4 mL of the pre-gel solution and 100 μL of a 4 × 10^6^ cells/mL suspension, and added to four wells of a 48-well plate (350 μL per well). Confocal images were captured using a Leica STELLARIS 5 confocal microscope. The acquired image data were thoroughly analyzed using LASX software, and a 3D structure with a depth of 150 μm (z-direction) was reconstructed from 220 optical slices in the x–y plane, resulting in a total volume of 581.25 × 581.25 × 150 μm³. Every 44 slices were compressed into a MIP image, and the number of cells in each MIP image was calculated using ImageJ software, yielding the total cell count within the annealed GH across the different culture durations. Each experiment was performed in quadruplicate.

*Construction of Tissue Engineered Skin*: A cylindrical PDMS mold was created, featuring an outer diameter of 22 mm, inner diameter of 12 mm, and height of 10 mm, along with a removable base of 12 mm diameter and 7 mm thickness. A PET membrane (12 mm diameter, 3 μm pore size) was placed on the base. Afterward, 350 μL of the GH/SH-HA/HDF cell composite was added and incubated at 37 °C for 30 min. After gelation, the assembly was inverted, the base was carefully removed using tweezers, and 500 μL of FbGrowth medium was added. The assembly was transferred to a 6-well plate containing 3 mL of FbGrowth medium for further culturing. After 3 d, HEKs were seeded on the PET membrane surface at a density of 5000 cells/mm^2^, with the medium replaced with KcGrowth. After another 3 d, the culture was switched to an air–liquid interface (ALI), and the medium was changed to EpiGrowth (Guangdong Biocell Biotechnology Co., Ltd., China) with daily replacement. Electrical resistance measurements of the engineered skin were conducted using a RE1600 cell resistance meter (Beijing Jinggong Hongtai Technology Co., Ltd., China) on days 0 (start of ALI), 7, and 14. Each experiment was performed in quadruplicate.

*Histological and Immunofluorescence Analysis of Engineered Skin:* Paraffin-embedded engineered skin samples were sectioned into 5 µm slices, followed by deparaffinization, hydration, and staining with hematoxylin and eosin (Yiermei Biotechnology Co., Ltd., China). For multiplex immunohistochemistry (mIHC) analysis, the sections were subjected to antigen retrieval after deparaffinization and hydration. Endogenous peroxidase activity was blocked using 3% hydrogen peroxide, washed with PBS, and blocked with 3% BSA (Shanghai Hushi Laboratory Equipment Co. Ltd., Shanghai, China). The sections were then incubated overnight with a primary antibody (CK10 Rabbit, Proteintech, USA) diluted in PBS at a ratio of 1:2000. An HRP-conjugated secondary antibody (MaxVision HRP-Polymer anti-Rabbit IHC Kit, Fuzhou Maixin Biotechnology Development Co., Ltd., China), matching the primary species, was then applied, followed by TSA staining with fluorescein tyramide (AAT Bioquest, USA). Antibodies were removed using a citrate antigen retrieval solution (Yiermei Biotechnology Co., Ltd., China), and nonspecific binding sites were blocked with 3% BSA before proceeding to the next round of fluorescence labeling. The same protocol was repeated using a secondary primary antibody (CK14 Rabbit, Proteintech, USA) in conjunction with Cy3 tyramide (AAT Bioquest, USA) for TSA staining. Finally, the sections were stained with freshly prepared DAPI and mounted for imaging. ZO-1 staining was performed using a primary antibody (ZO-1 Rabbit, Proteintech, USA) diluted in PBS at a ratio of 1:2000. Subsequently, a secondary antibody, Alexa Fluor 555 AffiniPure Donkey Anti-Rabbit IgG (H+L) (Jackson ImmunoResearch, USA), was applied at a 1:1000 dilution in PBS. Samples were incubated at room temperature for 1 h and washed with PBS. An autofluorescence quencher (Nanjing Yiermei Biotechnology Co., Ltd., China) was used to minimize autofluorescence. Finally, the nuclei were counterstained with DAPI before imaging.

*Treatment of Engineered Skins with Test Chemicals and Cell Viability:* After 14 d of ALI culturing, 80 μL of 5% sodium dodecyl sulfate (SDS; Shanghai Aladdin Biochemical Technology Co., Ltd., China), isopropanol (Meryer Co., Ltd., China), and PBS were applied to the surface of the engineered skin. After 15 min of exposure, the chemicals were washed away with PBS, and fresh EpiGrowth medium was added for a 42-hour incubation period. Subsequently, the medium was replaced with 1 mL of MTT solution for 4 h incubation. After incubation, the engineered skin was carefully removed using tweezers and transferred to a 2 mL EP tube and treated with 1 mL of solubilization solution (50 mL isopropanol + 1 mL of 2M HCl), followed by overnight incubation at 37 °C. Then, 100 μL of the solubilized solution was transferred to a 96-well plate, and the optical absorbance was measured at 570 nm using a microplate reader, with PBS serving as the negative control. Each test was conducted in triplicate.

*Statistical analysis:* Each experiment was conducted in at least triplicate (n ≥ 3). All data are presented as mean ± standard deviation. Statistical differences were analyzed using GraphPad Prism 5. An unpaired student’s t-test was used to calculate the statistical difference between two measurements, where statistical significance was set at P < 0.05.

**Supplementary Figures:**


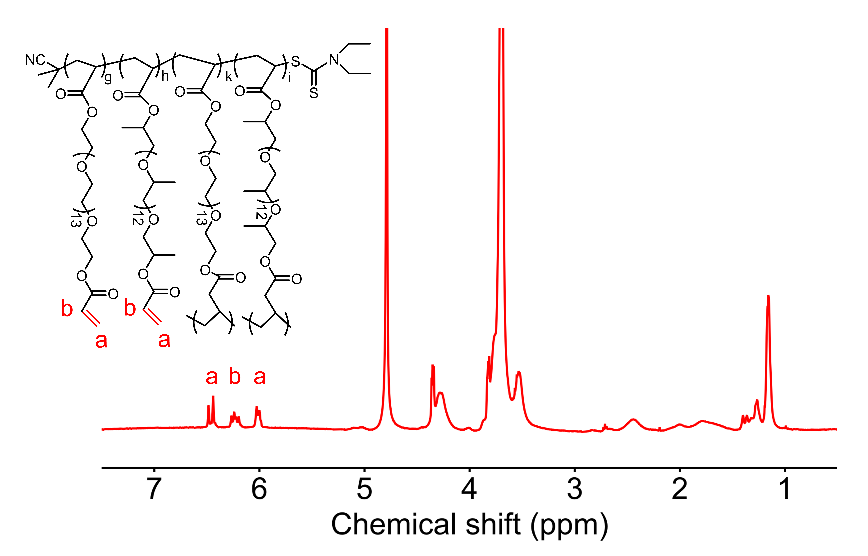


**Figure S1.** ^1^H NMR spectra of HB-PEG.


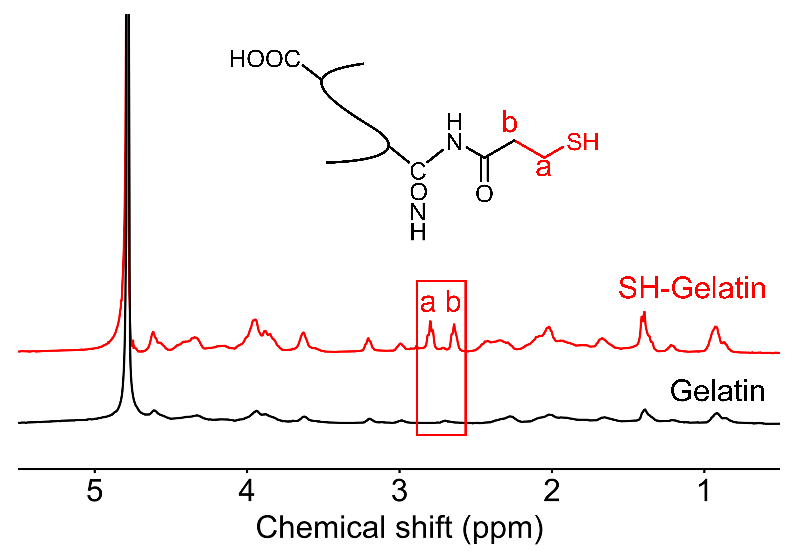


**Figure S2.** ^1^H NMR spectra of gelatin and thiolated gelatin (SH-Gelatin).


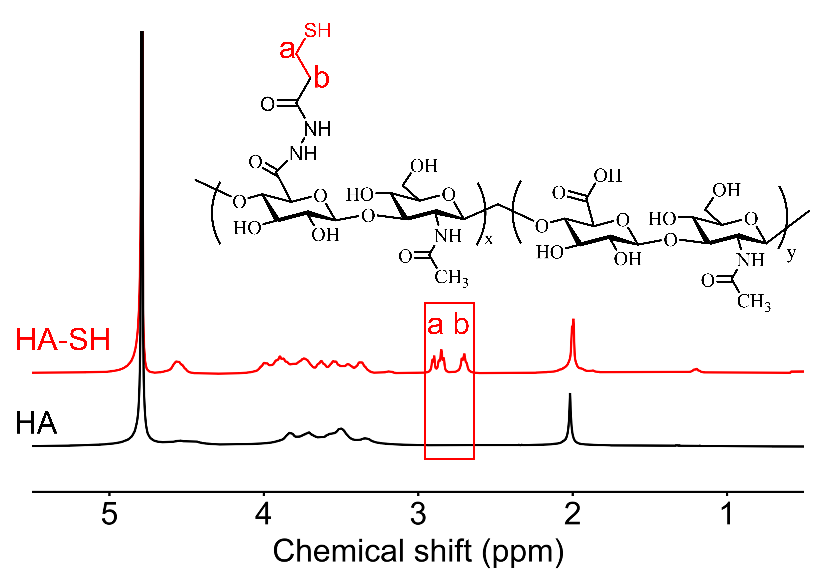


**Figure S3.** ^1^H NMR spectra of hyaluronic acid (HA) and thiolated hyaluronic acid (SH-HA).


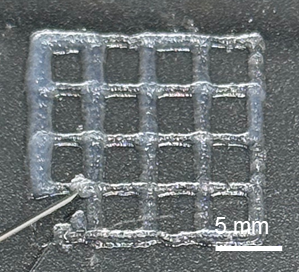


**Figure S4.** Images of the construct printed using densely packed HMPs alone


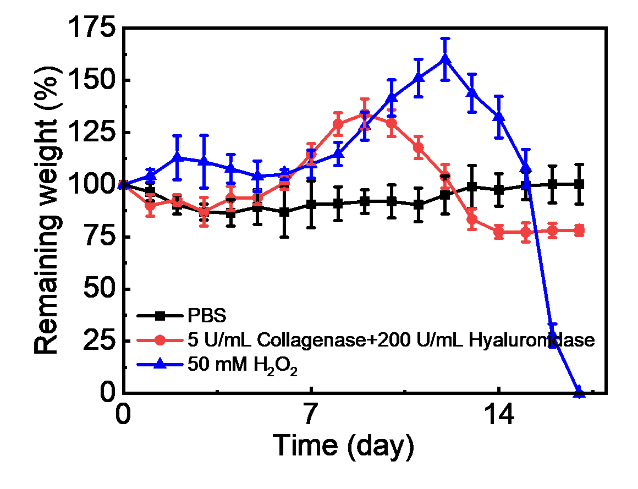


**Figure S5.** In vitro degradation profiles of annealed GH in PBS buffer, PBS buffer containing 5 U/mL collagenase and 200 U/mL hyaluronidase, and a 50 mM hydrogen peroxide solution.

The degradation experiment was conducted by measuring the relative change in the wet weight of the annealed GH compared to its initial weight at different time points. As shown in **Figure S5**, the annealed GH remains stable in PBS buffer, with no significant changes in volume (swelling or shrinkage), suggesting good stability. However, it undergoes an increase in mass (during the first 12 days) followed by a rapid decline (after day 12) when exposed to a high concentration of hydrogen peroxide, simulating reactive oxygen species (ROS) conditions. This is because, as degradation progresses, the hydrogel network gradually breaks down, resulting in a more porous and loosened structure, which facilitates water absorption and leads to an apparent increase in mass. As degradation further advances, the hydrogel structure eventually collapses and fully disperses into the medium, causing a sharp decline in mass at the later stages. Additionally, a similar situation occurs when the annealed GH is immersed in enzymatic environments, suggesting the gradual degradation of the material. These results indicate that the material we used is biodegradable. However, it is important to note that, given the actual concentrations of ROS and enzymes under standard culture conditions are significantly lower than those applied in our degradation experiments, the annealed GH maintains its structural integrity throughout the experimental period (two weeks).


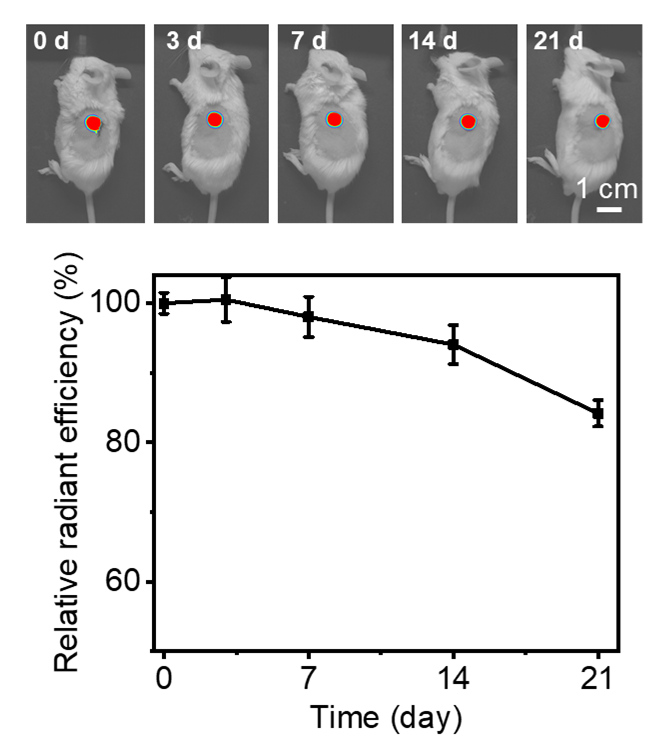


**Figure S6.** Representative in vivo pseudo-colored images of annealed GH following subcutaneous injection over three weeks, along with the corresponding cumulative fluorescence intensity curve.


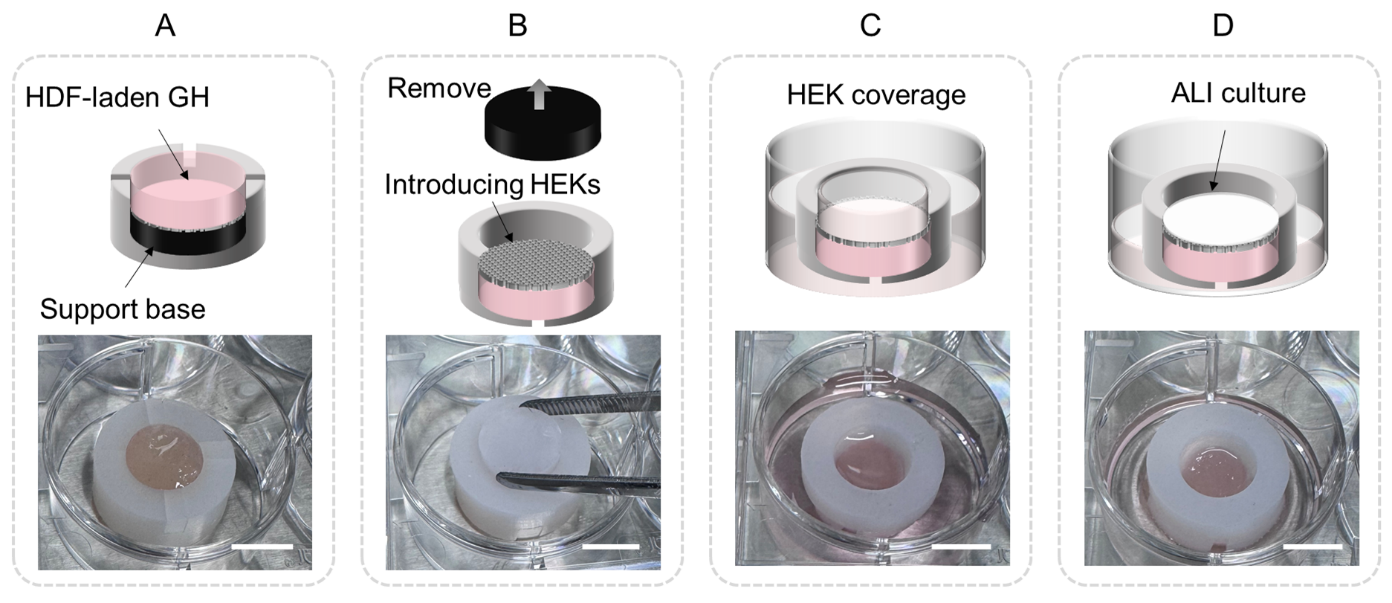


**Figure S7.** Construction of full-thickness skin within a PDMS mold: (a) Formation of the HDF-laden annealed GH-based dermal layer; (b) Inverting the mold and removing the bottom support base, followed by seeding HEK cells onto the upper part of the mold; (c) Fully immersing the mold in culture medium to allow HEK cells to proliferate and cover the surface; (d) Adjusting the medium volume to regulate the liquid level, enabling air-liquid interface (ALI) culture for HEK cells. Scale bar = 1 cm.


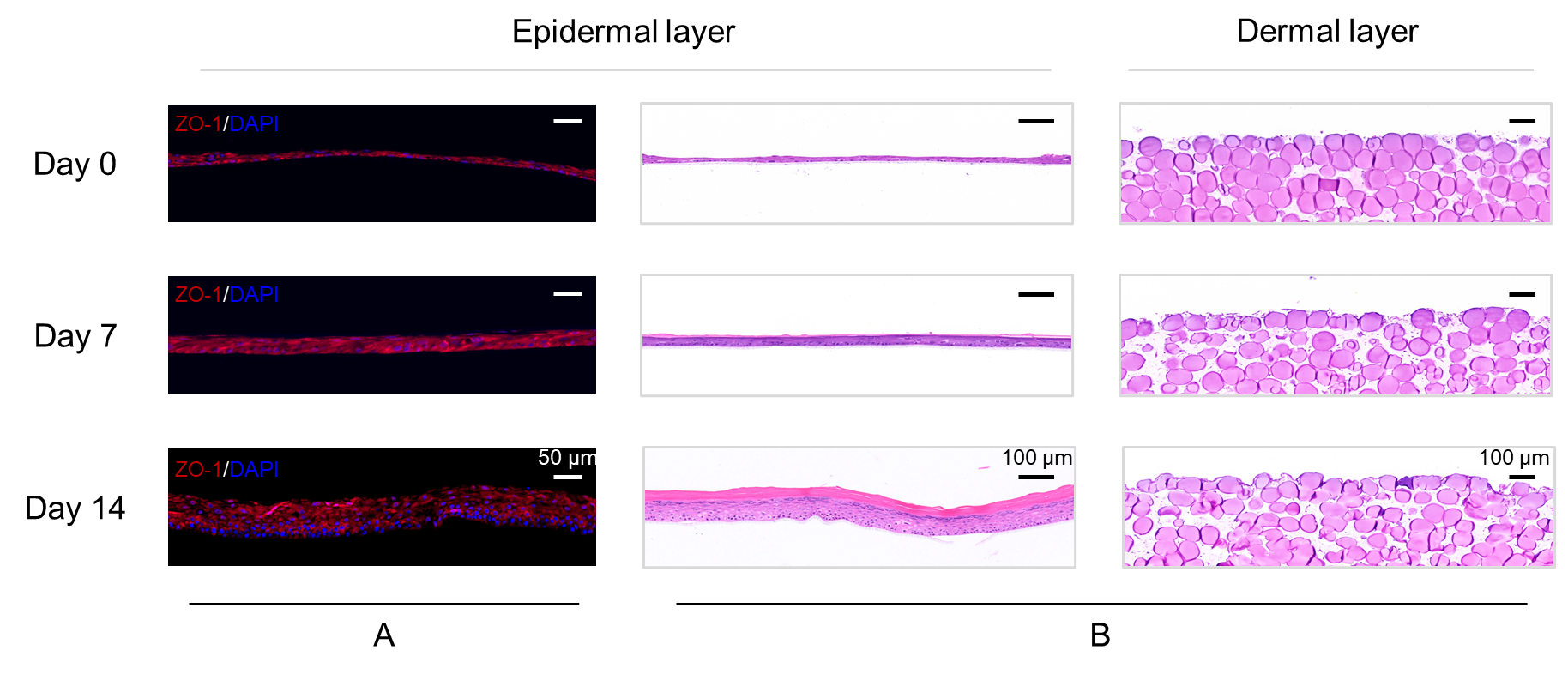


**Figure S8.** (a) ZO-1 fluorescence staining of the epidermal layer and HE staining of both the epidermal and dermal layers in full-thickness skin equivalent.

**Reference:**

[1] J. Zhang, H. Yong, Q. Xu, Y. Miao, J. Lyu, Y. Gao, M. Zeng, D. Zhou, Z. Yu, H. Tai, W. Wang, *Chem. Mater.* **2018**, *30*, 6091.

[2] X. Z. Shu, Y. Liu, Y. Luo, M. C. Roberts, G. D. Prestwich, *Biomacromolecules* **2002**, *3*, 1304.

[3] J. Zhang, X. Sun, Y. Heng, Y. Zeng, Y. Wang, Y. Shen, A. Peng, W. Tang, M. Zeng, Z. Yu, *ACS Appl. Mater. Interfaces* **2024**, *16*, 39784.
